# Skin-Whitening and Anti-Photoaging Activities of *Limosilactobacillus reuteri* DS0333 Culture Supernatant Produced in Unripe *Rubus occidentalis* L. Extract-Supplemented Medium

**DOI:** 10.4014/jmb.2606.06047

**Published:** 2026-10-08

**Authors:** Nu Ri Song, Seo Yeon Shin, Jae Hee Byun, Seon Ju Kim, Hye Young Kim, Won Seok Kim, Kyung Mok Park

**Affiliations:** 1 Department of Biocosmetics, Dongshin University, 185, Gunjae-ro, Naju 58245, Jeonnam, Republic of Korea; 2 RAPPOL, 30, Okcheon-gil, Suncheon 57941, Jeonnam, Republic of Korea; 3 Department of Petrochemical Process, Hanyeong University, 18-43, Janggunsan-gil, Yeosu 59720, Jeonnam, Republic of Korea

**Keywords:** *Limosilactobacillus reuteri*, *Rubus occidentalis*, Probiotics, Prebiotics, Synbiotics, Skin physiological activities

## Abstract

This study investigated the skin-whitening and anti-photoaging activities of a culture supernatant obtained by cultivating *Limosilactobacillus reuteri* DS0333 in chemically defined medium (CDM) supplemented with unripe *Rubus occidentalis* L. extract (UROE). DS0333 was cultured in UROE-supplemented medium, and the resulting DS0333/(CDM+UROE) group was evaluated alongside all control and comparison groups. The DS0333/(CDM+UROE) culture supernatant showed enhanced DPPH and ABTS radical scavenging activities compared with all other experimental groups. In α-MSH-stimulated B16F10 cells, the culture supernatant reduced melanin content and intracellular tyrosinase activity and downregulated MITF, TRP-1, and TRP-2 expression. In UVB-irradiated human dermal fibroblasts, the culture supernatant increased type I procollagen production, reduced MMP-1 levels, and suppressed MMP-1/MMP-3 expression through attenuation of the MAPK/AP-1 signaling pathway. These results suggest that culturing DS0333 in UROE-supplemented medium enhances skin-whitening and anti-photoaging activities, potentially through cultivation-associated modification of extracellular metabolites in the culture matrix. Therefore, the DS0333/(CDM+UROE) culture supernatant may serve as a promising multifunctional cosmetic ingredient for skin applications.

## Introduction

Skin aging is a multifactorial biological process driven by intrinsic chronological changes and extrinsic exposomal stressors, including ultraviolet (UV) radiation, air pollution, climate-related factors, and lifestyle-associated oxidative stress [1, 2]. Among these factors, UVB irradiation accelerates photoaging by increasing intracellular reactive oxygen species (ROS), activating mitogen-activated protein kinase (MAPK) signaling, and inducing activator protein-1 (AP-1)-dependent expression of matrix metalloproteinases (MMPs), which degrade dermal extracellular matrix components such as type I collagen [3, 4]. Therefore, inhibition of oxidative stress, MMP induction, and collagen degradation is a key strategy for developing functional anti-aging cosmetic ingredients. In addition to photoaging, hyperpigmentation is another major cosmetic concern associated with excessive melanin production. Melanogenesis is regulated primarily by tyrosinase, the rate-limiting enzyme in melanin biosynthesis, together with tyrosinase-related proteins such as TRP-1 and TRP-2 [5, 6]. These melanogenic enzymes are transcriptionally regulated by microphthalmia-associated transcription factor (MITF), which is activated in response to melanogenic stimuli such as α-melanocyte-stimulating hormone (α-MSH). Therefore, suppression of melanin accumulation, intracellular tyrosinase activity, and MITF/TRP-mediated signaling has been widely considered an effective strategy for developing skin-whitening cosmetic ingredients [7–9].

The skin microbiome has recently emerged as a functional regulator of skin barrier integrity, immune homeostasis, pigmentation responses, and cutaneous aging. Microbiome-derived and fermentation-derived approaches, including *Limosilactobacillus*-derived preparations, have been explored as topical or cosmetic ingredients because microbial cells, cell-derived components, and fermentation-associated metabolites can modulate oxidative stress, barrier function, inflammatory responses, and skin-related physiological activities [10–12]. In parallel, polyphenol-rich plant extracts, including berry-derived Rubus extracts, are attractive natural materials because their phenolic acids, flavonoids, and anthocyanins may contribute to antioxidant, anti-melanogenic, and collagen-protective activities [13, 14].

Although probiotic-derived preparations and plant extracts have individually been investigated for cosmetic applications, limited studies have evaluated whether cultivation of probiotic strains in plant extract-supplemented media can alter extracellular bioactivity and enhance skin-related functions compared with simple non-cultured mixtures. Therefore, this study aimed to determine whether cultivation of *L. reuteri* DS0333 in UROE-supplemented medium produces culture supernatants with enhanced skin-whitening and anti-photoaging activities compared with DS0333 culture alone, UROE alone, and a simple non-cultured mixture of the two components.

## Materials and Methods

### Chemicals and Reagents

All chemicals and reagents used in this study were of analytical grade unless otherwise stated. 2,2-Diphenyl-1-picrylhydrazyl (DPPH; Cat. No. D9132), 2,2′-azino-bis(3-ethylbenzothiazoline-6-sulfonic acid) diammonium salt (ABTS; Cat. No. A1888), potassium persulfate (Cat. No. P5592), α-melanocyte-stimulating hormone (α-MSH; Cat. No. M4135), 3,4-dihydroxy-L-phenylalanine (L-DOPA; Cat. No. D9628), arbutin (Cat. No. A4256), 4-phenylazobenzyloxycarbonyl-Pro-Leu-Gly-Pro-Arg (Cat. No. 89064), and dimethyl sulfoxide (DMSO; Cat. No. D8418) were purchased from Sigma-Aldrich (USA). Dulbecco’s modified Eagle’s medium (DMEM), fetal bovine serum (FBS), penicillin–streptomycin (P/S), phosphate-buffered saline (PBS), trypsin–EDTA, and other cell culture reagents used for B16F10 cell maintenance were purchased from Gibco (Thermo Fisher Scientific, USA). Fibroblast Medium (FM), fibroblast growth supplement (FGS), and penicillin–streptomycin solution used for human dermal fibroblast culture were obtained as components of the Fibroblast Medium Complete Kit (ScienCell Research Laboratories, USA; Cat. No. 2301). Type I procollagen production was quantified using a Procollagen Type I C-peptide (PIP) EIA Kit (Takara Bio Inc., Japan; Cat. No. MK101), and MMP-1 production was determined using a Human Pro-MMP-1 Quantikine ELISA Kit (R&D Systems, a Bio-Techne brand, USA; Cat. No. DMP100). Primary antibodies used for western blot analysis are listed in Table 3. Enhanced chemiluminescence detection reagent was obtained from Advansta (USA; Cat. No. K-12045-D50), and protein concentrations were determined using a Bradford assay reagent obtained from Bio-Rad (Bio-Rad Laboratories, USA; Cat. No. #5000205).

### Preparation of DS0333 Culture Supernatants and Experimental Groups

Unripe fruits of *Rubus occidentalis* L. were harvested in May from Gochang-gun, Jeollabuk-do, Republic of Korea. The harvested fruits were dried in a hot-air dryer (CF-21WF, Jeiotech, Republic of Korea) at 45°C for 24 h, sealed, and stored under refrigerated conditions until extraction. The dried fruits were extracted with 5% ethanol at a solid-to-solvent ratio of 1:20 (w/v) using a high-speed vacuum low-temperature extractor (Cosmos 660, Kyungseo Machine, Republic of Korea) for 4 h. The extract was filtered and concentrated under reduced pressure at 55°C and 50 mbar using a rotary vacuum concentrator (Rotavapor R-220, Switzerland), followed by lyophilization. The resulting powdered extract was designated as unripe *R. occidentalis* extract (UROE), sealed, and stored at 4°C until further use. The lactic acid bacterial strain *Limosilactobacillus reuteri* DS0333 (KCTC 12596BP) was obtained from the Korean Collection for Type Cultures. *L. reuteri* DS0333 was revived in De Man–Rogosa–Sharpe (MRS) medium under anaerobic conditions at 37°C for 24 h. The activated culture was transferred to chemically defined medium (CDM) and further incubated under the same conditions to obtain a starter culture. The starter culture was inoculated into CDM or UROE-supplemented CDM at 3% (v/v) and incubated anaerobically at 37°C for 24 h. After inoculation, the initial OD_600_ values were approximately 0.043 for DS0333/CDM and 0.049 for DS0333/(CDM+UROE). To distinguish the contributions of the basal medium, DS0333 culture supernatant, UROE, simple mixing, and cultivation of DS0333 in UROE-supplemented medium, five experimental conditions were established as shown in Table 1. For the UROE+CDM group, UROE was added to CDM at the designated concentration without bacterial inoculation and incubated under the same temperature and time conditions as the DS0333 culture samples. For the DS0333/CDM group, DS0333 was inoculated into CDM and incubated anaerobically at 37°C for 24 h. For the DS0333/(CDM+UROE) group, UROE was added to CDM before bacterial inoculation, and the final effective concentration of UROE during cultivation was 100 μg/mL. DS0333 was then inoculated into the UROE-supplemented CDM and incubated under the same culture conditions. Following incubation, the DS0333/CDM and DS0333/(CDM+UROE) cultures were autoclaved at 121°C for 15 min and centrifuged at 3,000 × g for 10 min. The resulting cell-free supernatants were collected in new tubes and stored at -70°C until further use. The (DS0333/CDM)+UROE group, representing the simple mixture, was prepared by mixing the DS0333/CDM culture supernatant with UROE+CDM immediately before treatment, without prior cultivation of DS0333 in the presence of UROE. This experimental design was used to determine whether the biological activity observed in the DS0333/(CDM+UROE) group was associated with cultivation of DS0333 in UROE-supplemented medium rather than with simple mixing of DS0333 culture supernatant and UROE.

The 5% ethanol extraction condition was adopted to maintain continuity with the previously established extraction procedure for unripe *R. occidentalis* [21]. The extract was subsequently concentrated under reduced pressure and lyophilized before UROE was added to the bacterial medium.

### Cell Culture

B16F10 mouse melanoma cells were obtained from the American Type Culture Collection (ATCC; USA) and maintained in Dulbecco’s modified Eagle’s medium (DMEM) supplemented with 10% fetal bovine serum (FBS) and 1% penicillin–streptomycin (P/S). Human dermal fibroblasts (HDFs) were obtained from ScienCell Research Laboratories (USA) and maintained in Fibroblast Medium supplemented with 2% FBS, 1% fibroblast growth supplement (FGS), and 1% P/S. Both cell types were cultured at 37°C in a humidified atmosphere containing 5% CO_2_.

### Growth Curve

To evaluate whether UROE affects the growth of DS0333, DS0333 was inoculated at 3% (v/v) into CDM or UROE-supplemented CDM and incubated under anaerobic static conditions at 37°C. After inoculation, the initial OD600 values were approximately 0.043 for DS0333/CDM and 0.049 for DS0333/(CDM+UROE). During cultivation, OD_600_ was measured every 2 h for 28 h. Growth curves were generated from triplicate measurements, and data are presented as mean ± SD.

### Antioxidant Activity

To evaluate DPPH radical scavenging activity, 0.2 mM DPPH solution was mixed with the sample and incubated for 30 min, followed by absorbance measurement at 515 nm. For ABTS radical scavenging activity, ABTS solution and potassium persulfate were mixed in equal volumes and reacted in the dark at room temperature for 24 h to generate ABTS radicals. The resulting solution was then diluted to an absorbance of 0.7 (±0.02) at 734 nm to prepare a working solution. The sample was then mixed with this working solution and incubated in the dark for 5 min before measuring the absorbance at 734 nm using a microplate reader.

### Total Phenolic and Flavonoid Contents

Total phenolic content (TPC) was determined using the Folin–Ciocalteu colorimetric method with slight modifications [15]. Briefly, gallic acid was prepared as a standard solution at 1 mg/mL and serially diluted to generate a calibration curve. Each standard solution or sample was added to a tube, followed by the addition of 10-fold diluted Folin–Ciocalteu reagent. After incubation at room temperature for 3 min, 7.5% sodium carbonate solution was added, and the reaction mixture was incubated in the dark at room temperature for 1 h. The reaction mixture was then transferred to a 96-well plate, and the absorbance was measured at 765 nm using a microplate reader. TPC was calculated from the gallic acid calibration curve and expressed as micrograms of gallic acid equivalents per milliliter of sample (μg GAE/mL). Total flavonoid content (TFC) was determined using the sodium nitrite–aluminum chloride colorimetric method with slight modifications [16]. Briefly, catechin or quercetin was prepared as a standard solution at 1 mg/mL and serially diluted to generate a calibration curve. Each standard solution or sample was mixed with 80% ethanol, followed by the addition of 5% sodium nitrite solution. After incubation at room temperature for 5 min, 10% aluminum chloride solution was added, and the mixture was incubated for an additional 5 min. Subsequently, 1 M sodium hydroxide solution was added, and the absorbance was measured at 510 nm using a UV–visible spectrophotometer. TFC was calculated from the standard calibration curve and expressed as micrograms of quercetin equivalents per milliliter of sample (μg QE/mL).

### HPLC Chromatographic Profiling

HPLC analysis was performed to qualitatively compare the chromatographic profiles of the five experimental groups: CDM, DS0333/CDM, UROE+CDM, (DS0333/CDM)+UROE, and DS0333/(CDM+UROE). Phenyllactic acid (PLA) was prioritized for preliminary investigation as one potential candidate reference compound based on its association with lactic acid bacterial cultivation and its previously reported anti-melanogenic activity in *L. reuteri* DS0333 culture broth [17]. The PLA standard and all sample solutions were filtered through a 0.22 μm membrane filter before analysis. PLA was used only as an analytical retention-time reference in the present qualitative HPLC analysis and was not considered a validated or sole chemical marker. The retention time of the PLA standard was compared with the chromatographic profiles of the experimental groups under the HPLC conditions summarized in Table 2. No validated quantitative determination of PLA was performed.

### Collagenase Inhibitory Activity

Each of the five experimental samples was added to the reaction mixture at a final concentration of 1% (v/v). The sample was mixed with 4-phenylazobenzyloxycarbonyl-Pro-Leu-Gly-Pro-Arg substrate solution, followed by the addition of collagenase. The reaction mixture was then incubated at 37°C for 20 min. The reaction was terminated by adding 6% citric acid. Subsequently, ethyl acetate was added to induce phase separation. The resulting supernatant was collected, and the absorbance was measured at 320 nm using a quartz cuvette.

### MTT Assay

Cell viability was evaluated using the MTT assay. B16F10 cells and HDFs were seeded in 96-well plates and allowed to attach for 24 h. The cells were then treated with the samples and incubated for 72 h under standard culture conditions. After incubation, MTT solution was added to each well at a final concentration of 0.5 mg/mL, followed by incubation for 3 h to allow formazan formation. The culture medium was then carefully removed, and the resulting formazan crystals were dissolved in DMSO. Absorbance was measured at 570 nm using a Multiskan Sky spectrophotometer (Thermo Fisher Scientific, USA). Cell viability was calculated relative to the untreated control group.

In the initial cytotoxicity assessment, B16F10 cells and HDFs were treated with each of the five experimental samples at a final concentration of 1% (v/v) for 72 h. To further characterize the cytotoxicity profile of DS0333/(CDM+UROE), additional concentration-response assays were performed at 0.5, 1, 2.5, and 5% (v/v) for 72 h. Concentrations that maintained cell viability at an acceptable level relative to the untreated control were considered non-cytotoxic and were used for subsequent concentration-response experiments.

### Melanin Content Assay

B16F10 melanoma cells were seeded and incubated to allow for cell attachment. Melanin synthesis was then induced by treating the cells with α-MSH, followed by the addition of the sample. The cells were further incubated for 72 h at 37°C in a 5% CO_2_ atmosphere. After incubation, the culture medium was removed, and the cells were harvested using trypsin-EDTA. The resulting cell suspension was centrifuged at 16,000 × g for 15 min to obtain a pellet. To solubilize the melanin, 1 N NaOH solution containing 10% DMSO was added to the pellet and incubated at 80°C for 1 h. Finally, the absorbance was measured at 475 nm using a microplate reader. For the five-group comparison, α-MSH-stimulated B16F10 cells were treated with each experimental sample at a final concentration of 1% (v/v) for 72 h. For the additional concentration-response analysis, α-MSH-stimulated B16F10 cells were treated with DS0333/(CDM+UROE) at 0.5, 1, 2.5, and 5% (v/v) within the non-cytotoxic concentration range determined by the MTT assay.

### Intracellular Tyrosinase Activity

B16F10 melanoma cells were seeded and incubated, followed by treatment with α-MSH to induce melanin synthesis. The cells were then treated with the sample and incubated for 72 h. For the five-group comparison, α-MSH-stimulated B16F10 cells were treated with each experimental sample at a final concentration of 1% (v/v). For the additional concentration-response analysis, cells were treated with DS0333/(CDM+UROE) at 0.5, 1, 2.5, and 5% (v/v). After removing the culture medium, the cells were lysed using PRO-PREP lysis buffer. The lysates were kept on ice and then centrifuged at 19,000 g for 5 min at 4°C. The resulting supernatant was collected for the enzymatic assay, with L-DOPA serving as the substrate. Protein concentration was quantified using the Bradford assay. Equal amounts of protein were mixed with the substrate, and the formation of dopachrome was measured at 475 nm using a microplate reader.

### Type I Procollagen Assay

For the five-group comparison, HDFs were seeded and cultured for 24 h, irradiated with UVB, and then treated with each experimental sample at a final concentration of 1% (v/v) for an additional 72 h. Subsequently, the culture medium was collected, and the supernatant was used for the experiment. The degree of Type I procollagen synthesis was quantified using the Procollagen Type I C-peptide (PIP) EIA Kit (Takara Bio Inc., Japan).

### MMP-1 Assay

Human dermal fibroblasts (HDFs) were seeded and cultured for 24 h, irradiated with UVB, and then incubated for an additional 72 h with the indicated samples. For the five-group comparison, cells were treated with each experimental sample at a final concentration of 1% (v/v). For the concentration-response analysis, cells were exposed to DS0333/(CDM+UROE) at 0.5, 1, 2.5, and 5% (v/v). After incubation, the culture medium was collected and centrifuged to obtain the supernatant. MMP-1 production was quantified using a Human Pro-MMP-1 Quantikine ELISA Kit (R&D Systems, USA) according to the manufacturer's instructions.

### Western Blot Analysis

For comparative western blot analysis, B16F10 melanoma cells stimulated with α-MSH and HDFs irradiated with UVB were seeded and treated with each of the five experimental samples at a final concentration of 1% (v/v) for the indicated time intervals. The cells were then lysed using a lysis buffer and centrifuged to collect the supernatant. Protein concentration was quantified via the Bradford assay. Equal amounts of protein were mixed with SDS loading buffer, denatured at 100°C for 10 min, and separated by SDS-PAGE. The proteins were then transferred onto a membrane, which was blocked with 5% skim milk for 1 h. After washing four times with TBST (10 min each), the membrane was incubated with the primary antibody at room temperature for 1 h. Following another washing step, the secondary antibody was incubated for 1 h. Finally, the expression of skin aging-related proteins was visualized using an enhanced chemiluminescence (ECL) western blotting detection kit.

### Statistical Analysis

All data were analyzed using IBM SPSS Statistics version 27.0 (IBM Corp., USA). Comparisons among multiple experimental groups were performed using one-way analysis of variance (ANOVA), followed by Duncan’s multiple range test for post hoc analysis. Differences between two groups were assessed using Student’s t-test. Symbols denoting statistical differences and their corresponding comparison groups are specified in each figure legend. Because quantitative synergy analysis was not conducted in the present study, the enhanced activity observed in the DS0333/(CDM+UROE) group was interpreted as cultivation-associated enhancement rather than definitive synergistic activity.

## Results

### UROE Promotes DS0333 Growth During Cultivation in Extract-Supplemented Medium

To determine whether UROE could affect the growth of DS0333, bacterial growth was monitored by measuring OD_600_ at 2-h intervals. Compared with DS0333 cultured in CDM alone, the UROE-supplemented culture showed increased growth kinetics during the cultivation period, indicating that components present in UROE may serve as growth-supporting substrates or metabolic factors for DS0333 (Fig. 1). This result suggests that UROE can support DS0333 growth under the present culture conditions. The enhanced bacterial growth observed in the presence of UROE also indicates that the extracellular culture environment may differ from that of DS0333 cultured in CDM alone, providing a rationale for subsequent comparison of the corresponding culture supernatants.

### DS0333/(CDM+UROE) Culture Supernatant Enhances Antioxidant Activity

The antioxidant activity of the experimental groups was evaluated using DPPH and ABTS radical scavenging assays. In the DPPH assay, the CDM control exhibited a radical scavenging activity of 19.2%, whereas DS0333/CDM and UROE+CDM showed increased activities of 44.6% and 42.4%, respectively. The simple mixture group, (DS0333/CDM)+UROE, exhibited a DPPH radical scavenging activity of 48.7%. Among the five experimental groups, DS0333/(CDM+UROE) showed the highest DPPH radical scavenging activity at 62.8% (Fig. 2A). The ABTS radical scavenging activity increased from 28.9% in the CDM control to 47.2% in the DS0333/CDM group and 43.1% in the UROE+CDM group. The simple mixture group showed an activity of 50.2%, whereas the DS0333/(CDM+UROE) group exhibited the highest activity among the five experimental groups, reaching 61.9% (Fig. 2B). Collectively, DS0333/(CDM+UROE) increased DPPH and ABTS radical scavenging activities beyond those observed with DS0333/CDM, UROE+CDM, and their simple mixture. These findings suggest that cultivation of DS0333 in UROE-supplemented CDM may contribute to enhanced antioxidant activity through cultivation-associated changes in extracellular components. To further examine whether the enhanced antioxidant activity was associated with changes in phenolic-related components, total phenolic content and total flavonoid content were measured in UROE+CDM and DS0333/(CDM+UROE). DS0333/(CDM+UROE) showed higher total phenolic and flavonoid contents than UROE+CDM, suggesting that cultivation of DS0333 in UROE-supplemented medium may increase or modify phenolic- and flavonoid-related extracellular components (Fig. 2C).

### Comparative HPLC Chromatographic Profiling of the Five Experimental Groups

PLA was preliminarily investigated as one potential candidate reference compound among the metabolites that may be associated with DS0333 cultivation. This preliminary selection was based on its association with lactic acid bacterial cultivation and its previously reported anti-melanogenic activity in DS0333 culture broth [17]. Comparative HPLC profiling was performed for all five experimental groups: CDM, DS0333/CDM, UROE+CDM, (DS0333/CDM)+UROE, and DS0333/(CDM+UROE). The PLA standard produced a distinct peak at its characteristic retention time under the present chromatographic conditions (Fig. 3A). The five experimental groups exhibited qualitative differences in their chromatographic patterns, including differences in peak distribution within the 6–12 min retention-time range (Fig. 3B–3F). In particular, the chromatographic profile of DS0333/(CDM+UROE) differed from those of CDM, DS0333/CDM, UROE+CDM, and the simple mixture group. However, the altered peaks were not structurally identified, and the correspondence of a sample peak with the retention time of the PLA standard alone was insufficient to confirm its chemical identity. Moreover, PLA was neither quantitatively determined nor demonstrated to be selectively enriched in DS0333/(CDM+UROE). Therefore, the present qualitative HPLC results indicate cultivation-associated differences among the chromatographic profiles but do not establish PLA as a validated chemical marker or as the compound responsible for the enhanced biological activities.

### Cytotoxicity Evaluation of Experimental Groups in B16F10 cells and HDFs

Before evaluating the skin-whitening and anti-photoaging activities, the cytotoxicity of the experimental groups was examined using the MTT assay in B16F10 melanoma cells and human dermal fibroblasts. In B16F10 cells, all experimental groups maintained cell viability above approximately 94% of the untreated control, with CDM, DS0333/CDM, UROE+CDM, (DS0333/CDM)+UROE, and DS0333/(CDM+UROE) showing no marked reduction in cell viability (Fig. 4a). Similarly, in HDFs, cell viability remained above approximately 95% across all treatment groups (Fig. 4B). These results indicate that the tested samples did not exert notable cytotoxic effects under the present experimental conditions.

### DS0333/(CDM+UROE) Culture Supernatant Suppresses Melanogenesis in α-MSH-Stimulated B16F10 Cells

To evaluate the skin-whitening potential of the DS0333/(CDM+UROE) culture supernatant, melanogenesis-related endpoints were examined in α-MSH-stimulated B16F10 melanoma cells. Among the experimental groups, CDM, DS0333/CDM, UROE+CDM, and the simple mixture group, (DS0333/CDM)+UROE, reduced melanin content to 77.2%, 63.8%, 53.6%, and 52.9%, respectively. Notably, DS0333/(CDM+UROE) showed the lowest melanin content at 45.9%, representing a 64.0% reduction compared with the α-MSH-treated group and a further reduction compared with the simple mixture group (Fig. 5A). Intracellular tyrosinase activity was further measured to determine whether the reduction in melanin content was associated with inhibition of the rate-limiting melanogenic enzyme. DS0333/(CDM+UROE) strongly inhibited α-MSH-induced tyrosinase activity, reducing it to 108.4%, which corresponds to a 78.2% decrease compared with the α-MSH-treated group (Fig. 5B). Western blot analysis was performed to further investigate melanogenesis-related molecular mechanisms. α-MSH stimulation increased the expression of MITF and tyrosinase-related proteins, including TRP-1 and TRP-2. Treatment with the DS0333/(CDM+UROE) culture supernatant downregulated these melanogenesis-associated proteins. These results suggest that the DS0333/(CDM+UROE) culture supernatant suppresses melanogenesis by reducing melanin accumulation, inhibiting intracellular tyrosinase activity, and modulating the MITF/TRP melanogenic pathway (Fig. 5C-5F).

### DS0333/(CDM+UROE) Culture Supernatant Attenuates UVB-Induced Photoaging Responses in HDFs

To evaluate the anti-photoaging potential of the experimental groups, collagen metabolism-related endpoints were examined. First, collagenase inhibitory activity was measured using a synthetic collagenase substrate. Among the experimental groups, DS0333/(CDM+UROE) showed the highest collagenase inhibitory activity, suggesting that culture of DS0333 in UROE-supplemented medium may alter extracellular components associated with collagenase inhibitory activity capable of protecting dermal collagen from enzymatic degradation (Fig. 6A). The effect of the samples on collagen synthesis was then evaluated by measuring type I procollagen production in UVB-irradiated HDFs. UVB exposure markedly reduced type I procollagen levels compared with the non-irradiated control, indicating impaired collagen synthesis under photoaging stress. Treatment with DS0333/CDM, (DS0333/CDM)+UROE, and DS0333/(CDM+UROE) significantly restored type I procollagen production (Fig. 6B). MMP-1 production was also assessed as a representative marker of collagen degradation in UVB-induced photoaging. Treatment with DS0333/(CDM+UROE) significantly reduced UVB-induced MMP-1 production, and this inhibitory effect was stronger than that observed in the (DS0333/CDM)+UROE simple mixture group (Fig. 6C). Together, these findings indicate that DS0333/(CDM+UROE) may attenuate UVB-induced photoaging responses mainly by suppressing collagen-degrading activity.

### DS0333/(CDM+UROE) Culture Supernatant Modulates UVB-Induced Photoaging-Related Signaling Pathways

To investigate the molecular mechanism underlying the anti-photoaging activity of the DS0333/(CDM+UROE) culture supernatant, western blot analysis was performed in UVB-irradiated HDFs. UVB irradiation increased the expression of MMP-1 and MMP-3, which are representative matrix metalloproteinases involved in collagen degradation. Treatment with DS0333/CDM and UROE+CDM reduced these protein levels to some extent, whereas DS0333/(CDM+UROE) showed the strongest suppressive effect. The DS0333/(CDM+UROE) group also showed stronger inhibition than the (DS0333/CDM)+UROE simple mixture group, supporting the contribution of cultivation in UROE-supplemented medium to the anti-photoaging response (Fig. 7A-7C). The MAPK/AP-1 signaling pathway was further examined because it plays a key role in UVB-induced MMP expression and collagen degradation. UVB irradiation increased the phosphorylation of MAPK proteins, including ERK, JNK, and p38, as well as AP-1 components c-Fos and c-Jun. Treatment with DS0333/(CDM+UROE) attenuated the phosphorylation of these signaling proteins (Fig. 7D-7J). These results suggest that the anti-photoaging effect of the DS0333/(CDM+UROE) culture supernatant is associated with suppression of the MAPK/AP-1 pathway, leading to reduced MMP expression and improved collagen homeostasis.

### Concentration-Response Effects of DS0333/(CDM+UROE)

To further characterize the biological activity of DS0333/(CDM+UROE) and establish its cytotoxicity profile across the concentration range used in the functional assays, additional concentration-response experiments were performed at 0.5, 1, 2.5, and 5% (v/v). In B16F10 melanoma cells, cell viability remained high throughout the tested concentration range, indicating no marked cytotoxicity under the present experimental conditions (Fig. 8A). In α-MSH-stimulated B16F10 cells, melanin content decreased progressively with increasing concentrations of DS0333/(CDM+UROE), with the greatest reduction observed at 5% (v/v) (Fig. 8B). Intracellular tyrosinase activity also decreased significantly and progressively across the same concentration range, approaching the untreated control level at 5% (v/v) (Fig. 8C).

Similarly, DS0333/(CDM+UROE) did not cause marked cytotoxicity in HDFs across the tested concentration range (Fig. 8d). In UVB-irradiated HDFs, type I procollagen and MMP-1 production were further evaluated as representative photoaging-related endpoints. DS0333/(CDM+UROE) increased type I procollagen production and reduced MMP-1 production within the confirmed non-cytotoxic concentration range. However, these responses were not strictly monotonic across all tested concentrations (Fig. 8E and 8F).

Collectively, these additional experiments demonstrate that DS0333/(CDM+UROE) exerts anti-melanogenic and anti-photoaging activities across a confirmed non-cytotoxic concentration range. In particular, the progressive reductions in melanin content and intracellular tyrosinase activity support a concentration-responsive anti-melanogenic effect. However, because the type I procollagen and MMP-1 responses were not strictly concentration proportional, the overall findings are interpreted as concentration-response effects rather than evidence of a uniformly linear dose-dependent relationship across all endpoints.

## Discussion

This study demonstrates that the culture supernatant obtained after cultivation of DS0333 in UROE-supplemented CDM enhances multiple skin physiological activities, including antioxidant capacity, collagenase inhibition, melanogenesis suppression, and UVB-induced photoaging protection. The experimental design compared five conditions—CDM, DS0333/CDM, UROE+CDM, a simple non-cultured mixture, and DS0333/(CDM+UROE)—allowing the contribution of the culture process in the presence of UROE to be distinguished from the independent effects of DS0333 and UROE. The stronger activity of DS0333/(CDM+UROE) compared with the simple mixture suggests that cultivation-associated modification of extracellular components during DS0333 growth in UROE-supplemented medium may contribute to the observed bioactivity. This interpretation is consistent with previous evidence that microbial culture-derived preparations and fermentation-associated materials can improve skin-related endpoints, including barrier function and antioxidant capacity [18–20].

The enhanced activity observed in the DS0333/(CDM+UROE) group may be associated with cultivation-induced modification of extracellular metabolites or plant-derived constituents during DS0333 growth in UROE-supplemented medium. UROE contains phenolic compounds, including anthocyanins and flavonoids, with reported antioxidant and skin-protective properties [21–23]. Accordingly, the qualitative chromatographic differences observed in DS0333/(CDM+UROE) may reflect modification of DS0333-derived metabolites, transformation of UROE-derived constituents, or changes in the extracellular culture matrix. However, the present HPLC analysis cannot determine whether the altered peaks represent newly generated microbial metabolites, transformed phytochemicals, or differences originating from the culture matrix because the major peaks were neither structurally identified nor quantitatively validated. PLA was prioritized for preliminary investigation as one potential candidate reference compound based on its association with lactic acid bacterial cultivation and its previously reported anti-melanogenic activity in *L. reuteri* DS0333 culture broth [17]. Nevertheless, the present results did not demonstrate selective enrichment of PLA, and the enhanced biological activities cannot be attributed to PLA alone. Comprehensive LC–MS/MS-based targeted and untargeted metabolomic analyses are therefore required to identify metabolites that are generated, enriched, depleted, or transformed during DS0333 cultivation in UROE-supplemented medium. Multiple candidate marker compounds should subsequently be prioritized based on validated quantitative analysis, reproducibility across independent culture batches, and their associations with the observed antioxidant, anti-melanogenic, and anti-photoaging activities. Further functional validation using isolated compounds, standard-spiking experiments, or bioactivity-guided fractionation will also be required to establish causal relationships between specific metabolites and the observed biological effects.

The concentration-response experiments further demonstrated that DS0333/(CDM+UROE) reduced melanin content and intracellular tyrosinase activity across the tested non-cytotoxic concentration range, supporting its anti-melanogenic potential. Consistent with these functional findings, western blot analysis at 1% (v/v) showed reduced expression of MITF, TRP-1, and TRP-2. However, because these melanogenesis-related proteins were evaluated at only a single concentration, the present data do not establish concentration-dependent regulation of the MITF/TRP pathway. Reliable quantitative assessment across multiple concentrations would require additional independent experiments and optimization of the western blot conditions. Therefore, the current protein-expression results should be interpreted as preliminary mechanistic support for the observed anti-melanogenic effects rather than definitive evidence of a concentration-dependent molecular mechanism.

The anti-photoaging activity observed in HDFs is biologically plausible because UVB irradiation activates ROS-sensitive MAPK signaling and AP-1 transcriptional activity, leading to increased expression of MMPs and subsequent collagen degradation [24–27]. In the present study, DS0333/(CDM+UROE) increased type I procollagen production and decreased MMP-1 levels, and western blot data suggested suppression of the MAPK/AP-1 axis. Therefore, the DS0333/(CDM+UROE) culture supernatant may preserve dermal extracellular matrix homeostasis by simultaneously promoting collagen precursor synthesis and reducing collagen-degrading enzyme expression.

Interestingly, the simple mixture group showed slightly higher procollagen production than the DS0333/(CDM+UROE) group, suggesting that collagen synthesis and MMP suppression may be regulated through partially distinct mechanisms under the current in vitro conditions. While cultivation-associated changes may preferentially enhance anti-collagenolytic activity, direct stimulation of procollagen synthesis may depend on different extracellular components.

A critical point is that the current data demonstrate enhanced activity under UROE-supplemented cultivation conditions, but quantitative synergy was not directly assessed. Additional concentration-dependent interaction analyses using established synergy models will therefore be required to determine whether true synergistic interactions occur. Accordingly, the observed effects should currently be interpreted as culture condition-associated enhancement rather than definitive synergy.

The present study also cannot establish whether these effects are unique to DS0333 because no other bacterial strain was tested. Microbial conversion of plant phenolics and the resulting bioactivities can depend on both the plant matrix and the metabolic capacity of the individual strain. Accordingly, the present findings should be interpreted as evidence for the tested DS0333–UROE cultivation system under the specified conditions, rather than proof of DS0333 exclusivity or a universal effect of lactic acid bacterial cultivation. Comparative cultivation using additional strains and other lactic acid bacterial species, coupled with untargeted metabolomic profiling and targeted metabolite quantification, is needed to determine strain specificity and identify the metabolites associated with the enhanced biological activities.

The 5% ethanol extraction condition was adopted from a previously established procedure for unripe *Rubus occidentalis* [21]. That study compared fruit maturity rather than extraction solvents, and the present study did not compare alternative solvent systems. Ethanol was substantially removed during reduced-pressure concentration and lyophilization, although residual ethanol was not quantified. Therefore, 5% ethanol should be regarded as a literature-based, experimentally consistent condition rather than an optimized solvent system. Because solvent composition can alter the UROE phytochemical profile and substrates available for microbial transformation, future studies should compare extraction solvents and relate compositional differences to microbial metabolism and bioactivity.

## Conclusion

Cultivation of DS0333 in UROE-supplemented CDM produced a culture supernatant with enhanced antioxidant, anti-melanogenic, and anti-photoaging activities compared with the individual component and simple mixture groups. The observed effects may be associated with cultivation-induced modification of extracellular components during DS0333 growth in the presence of UROE. Furthermore, concentration-response analyses of DS0333/(CDM+UROE) demonstrated biological activity across a confirmed non-cytotoxic concentration range, strengthening the experimental basis for its skin-whitening and anti-photoaging potential. These findings suggest that cultivation of DS0333 in UROE-supplemented medium may represent a promising strategy for generating multifunctional culture-derived cosmetic materials with skin-protective potential.

## Figures and Tables

**Fig. 1:**
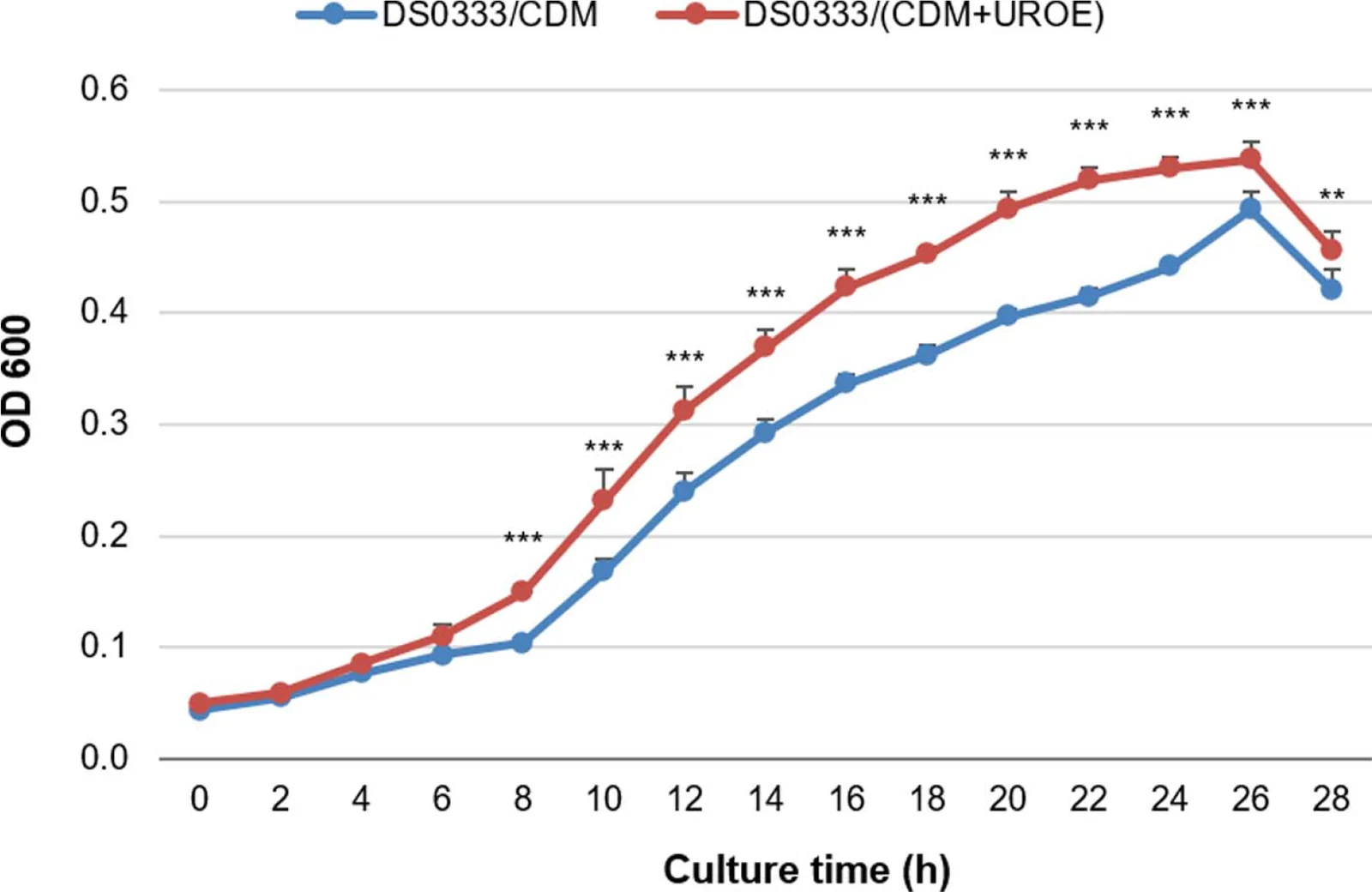
Growth kinetics of DS0333 cultivated in CDM with or without UROE. The growth of DS0333 in CDM (DS0333/CDM) and UROE-supplemented CDM [DS0333/(CDM+UROE)] was monitored by measuring the optical density at 600 nm (OD_600_) during anaerobic cultivation at 37°C. Data are presented as the mean ± SD of three independent experiments. Statistical differences between the two groups at each time point were evaluated using Student’s t-test. **p < 0.01 and ***p < 0.001 versus the DS0333/CDM group.

**Fig. 2:**
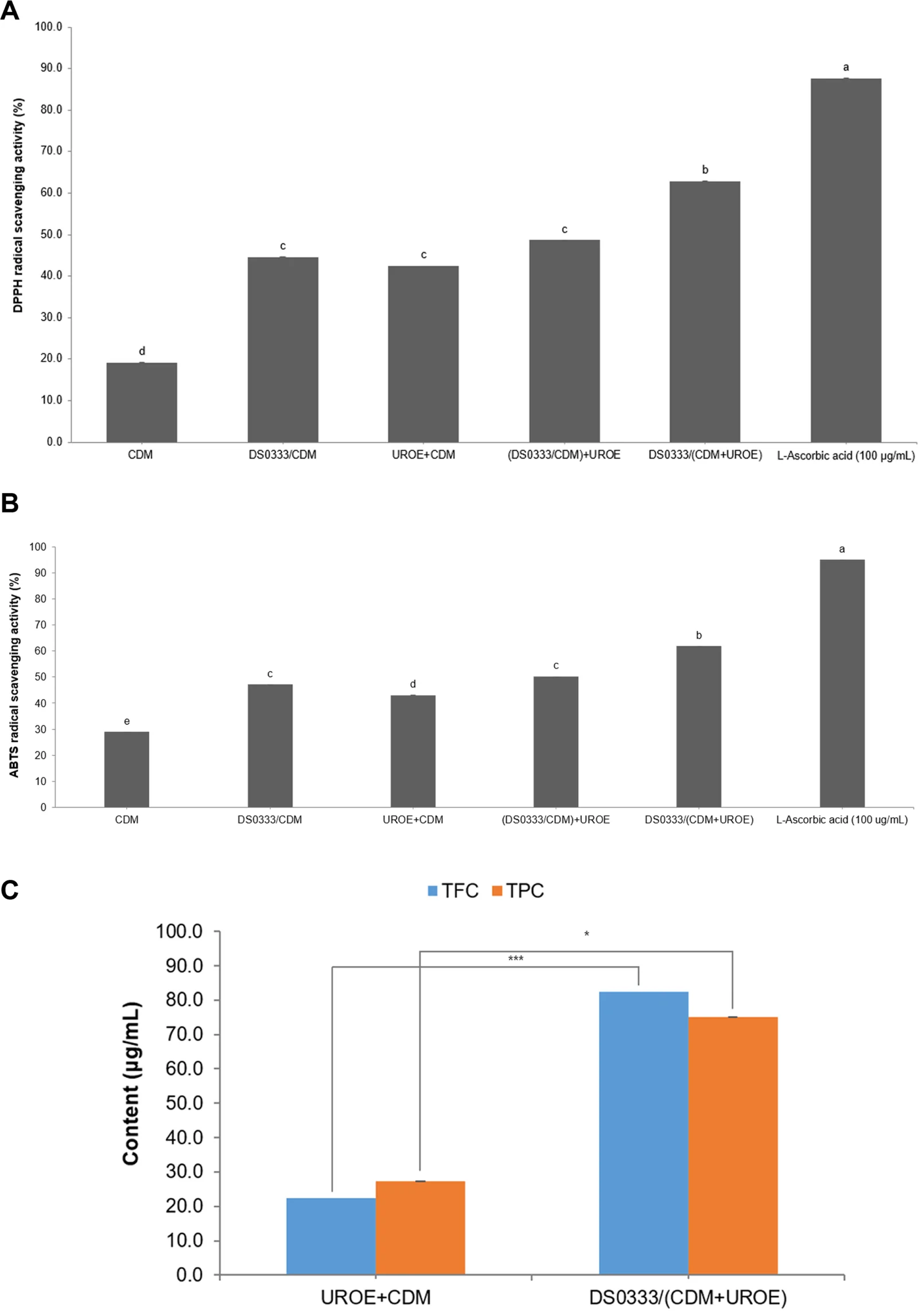
Antioxidant activities and phenolic and flavonoid contents of the experimental samples. (**A**) DPPH radical-scavenging activity (**B**) ABTS radical-scavenging activity. L-Ascorbic acid (100 μg/mL) was used as a positive control. (**C**) Total flavonoid content (TFC) and total phenolic content (TPC) of UROE+CDM and DS0333/(CDM+UROE). Data are presented as the mean ± SD of three independent experiments. For panels (**A**) and (**B**), statistical differences were analyzed using one-way ANOVA followed by Duncan’s multiple range test; values labeled with different lowercase letters are significantly different (*p* < 0.05). For panel (c), differences between UROE+CDM and DS0333/(CDM+UROE) were analyzed using Student’s t-test. *p < 0.05 and ***p < 0.001.

**Fig. 3:**
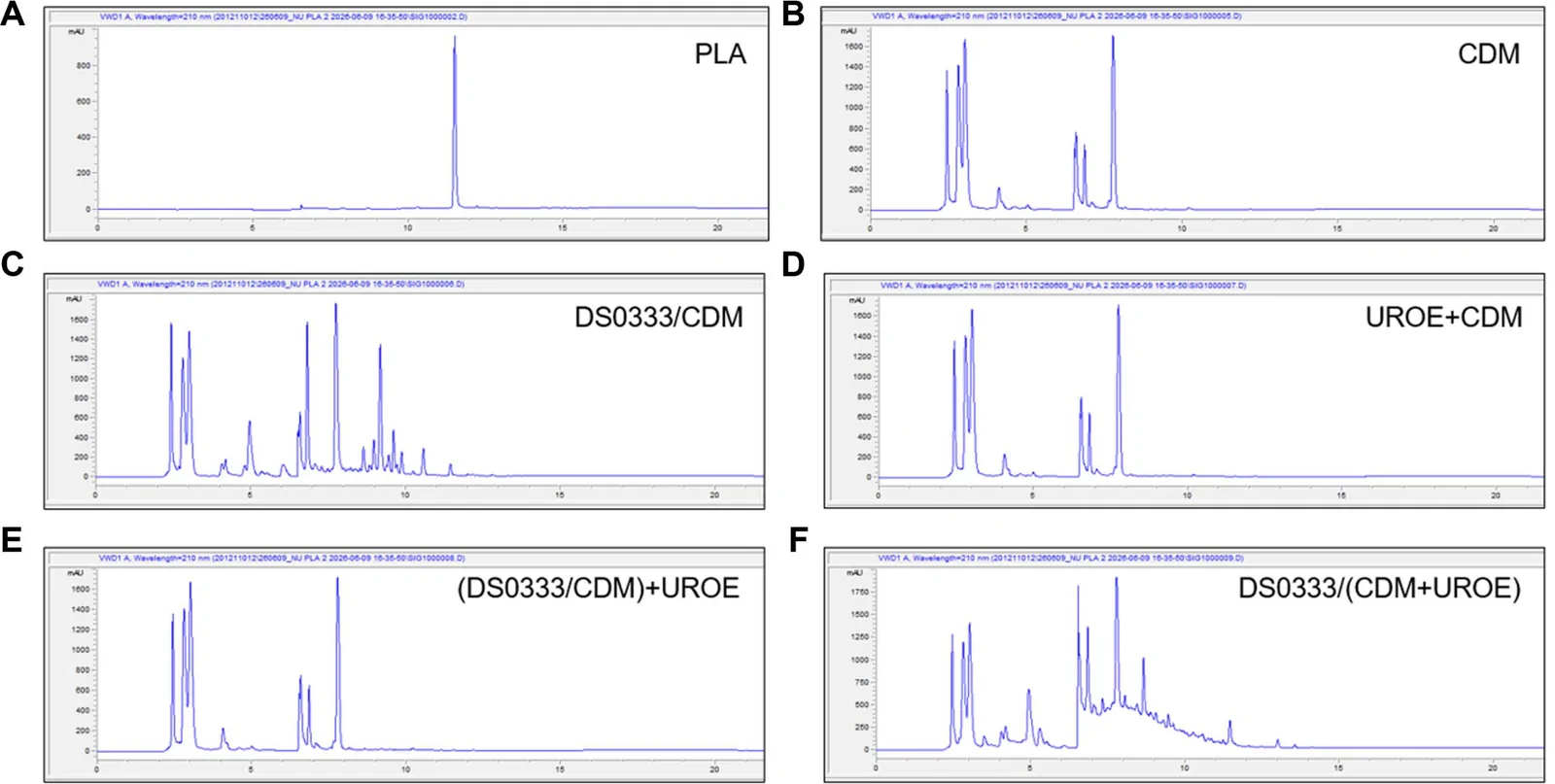
Comparative HPLC chromatographic profiles of PLA and the five experimental groups. (**A**) PLA analytical reference standard, (**B**) CDM, (**C**) DS0333/CDM, (**D**) UROE+CDM, (**E**) (DS0333/CDM)+UROE, and (**F**) DS0333/(CDM+UROE). PLA was used only as an analytical retention-time reference. The chromatograms were qualitatively compared, and PLA and the other peaks were not quantitatively determined.

**Fig. 4:**
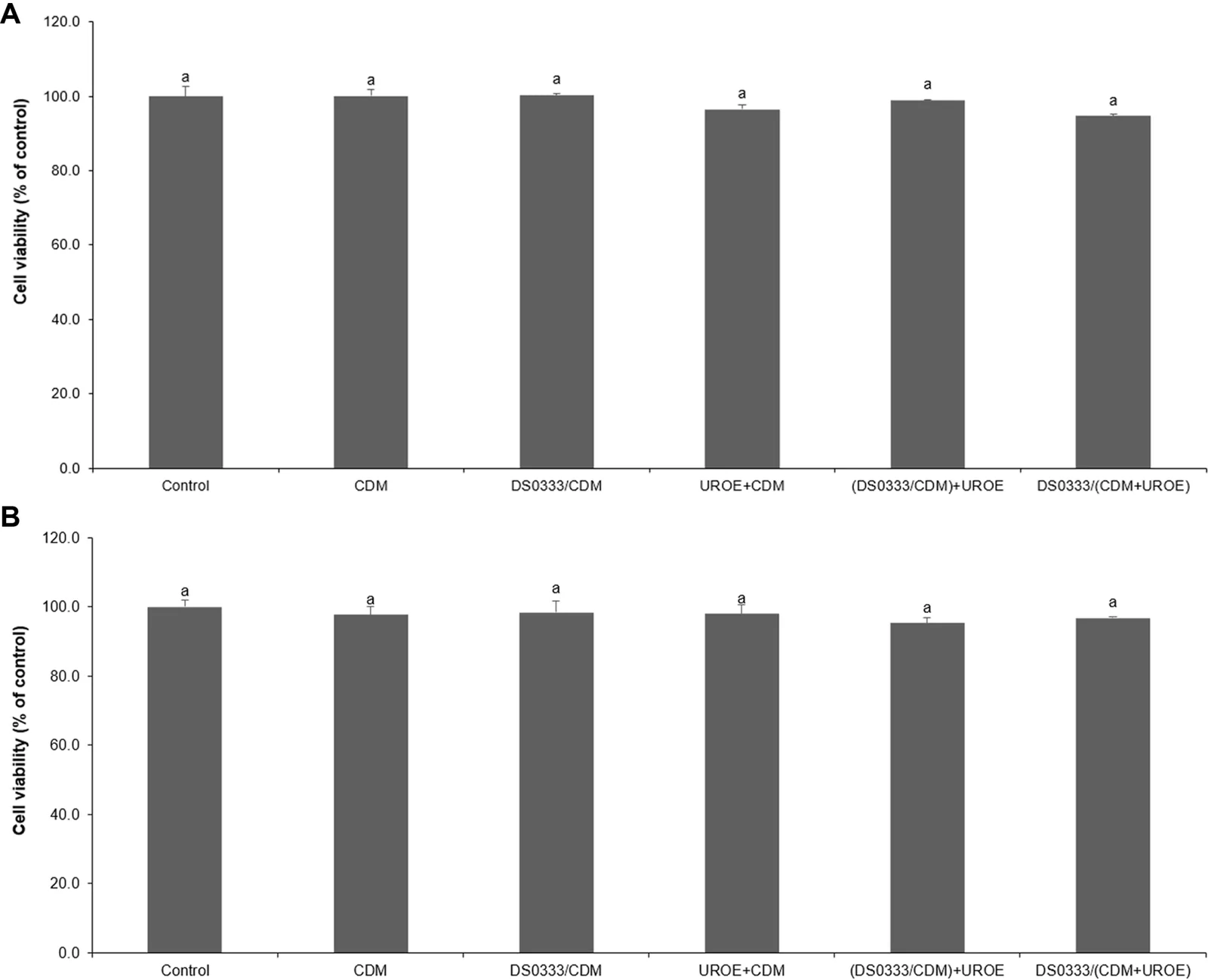
Effects of the experimental samples on cell viability. Cell viability was evaluated in (**A**) B16F10 melanoma cells and (**B**) HDFs. Untreated cells were used as the control, and their viability was set to 100%. Data are presented as the mean ± SD of three independent experiments. Statistical differences were analyzed using one-way ANOVA followed by Duncan’s multiple range test. Values labeled with the same lowercase letter are not significantly different (*p* ≥ 0.05).

**Fig. 5:**
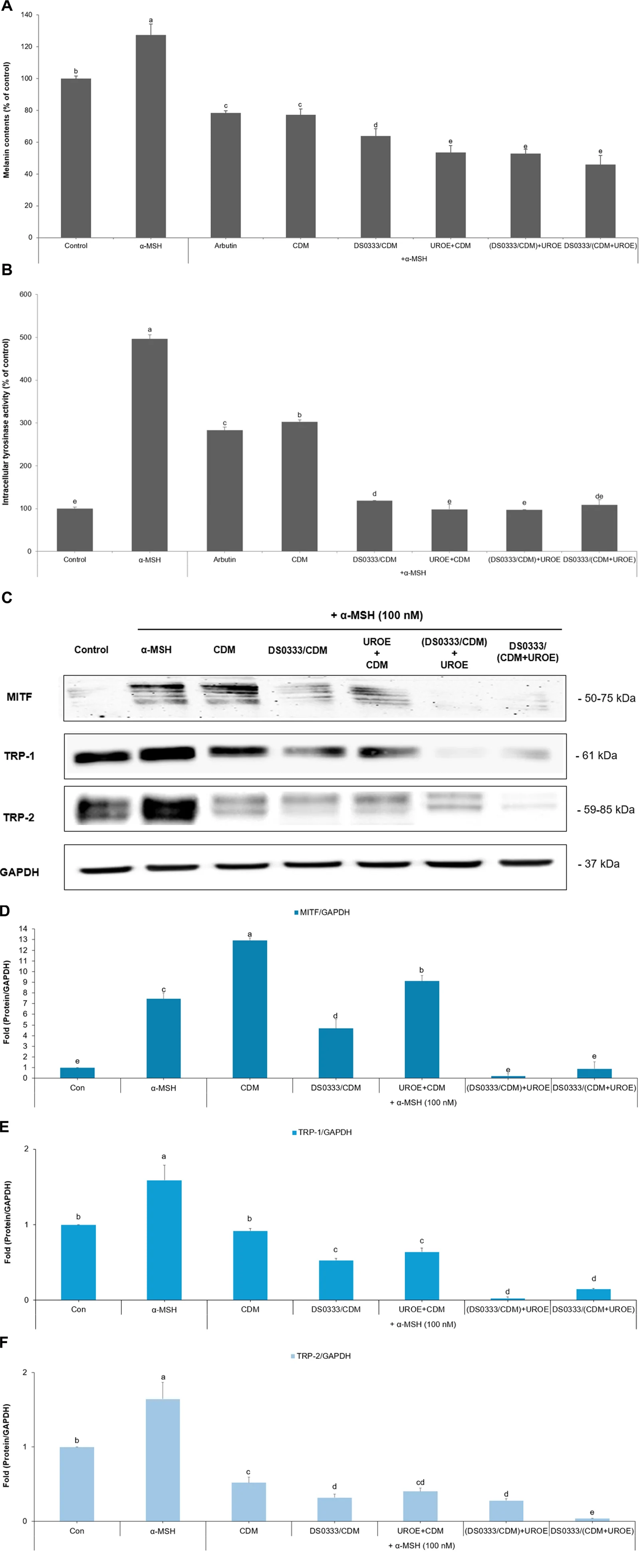
Effects of the experimental samples on α-MSH-induced melanogenesis in B16F10 melanoma cells. Cells were stimulated with α-melanocyte-stimulating hormone (α-MSH; 100 nM). Arbutin (200 μM) was used as a positive control. (**A**) Melanin content and (**B**) intracellular tyrosinase activity were measured after 72 h of treatment. (**C**) Protein expression levels of microphthalmia-associated transcription factor (MITF), tyrosinase-related protein 1 (TRP-1), and tyrosinase-related protein 2 (TRP-2) were determined by western blotting. GAPDH was used as the loading control. Densitometric quantification of (**D**) MITF, (**E**) TRP-1, and (**F**) TRP-2 protein expression was normalized to GAPDH and expressed relative to the untreated control. Data are presented as the mean ± SD of three independent experiments. Statistical differences were analyzed using one-way ANOVA followed by Duncan’s multiple range test. Values labeled with different lowercase letters are significantly different (*p* < 0.05).

**Fig. 6:**
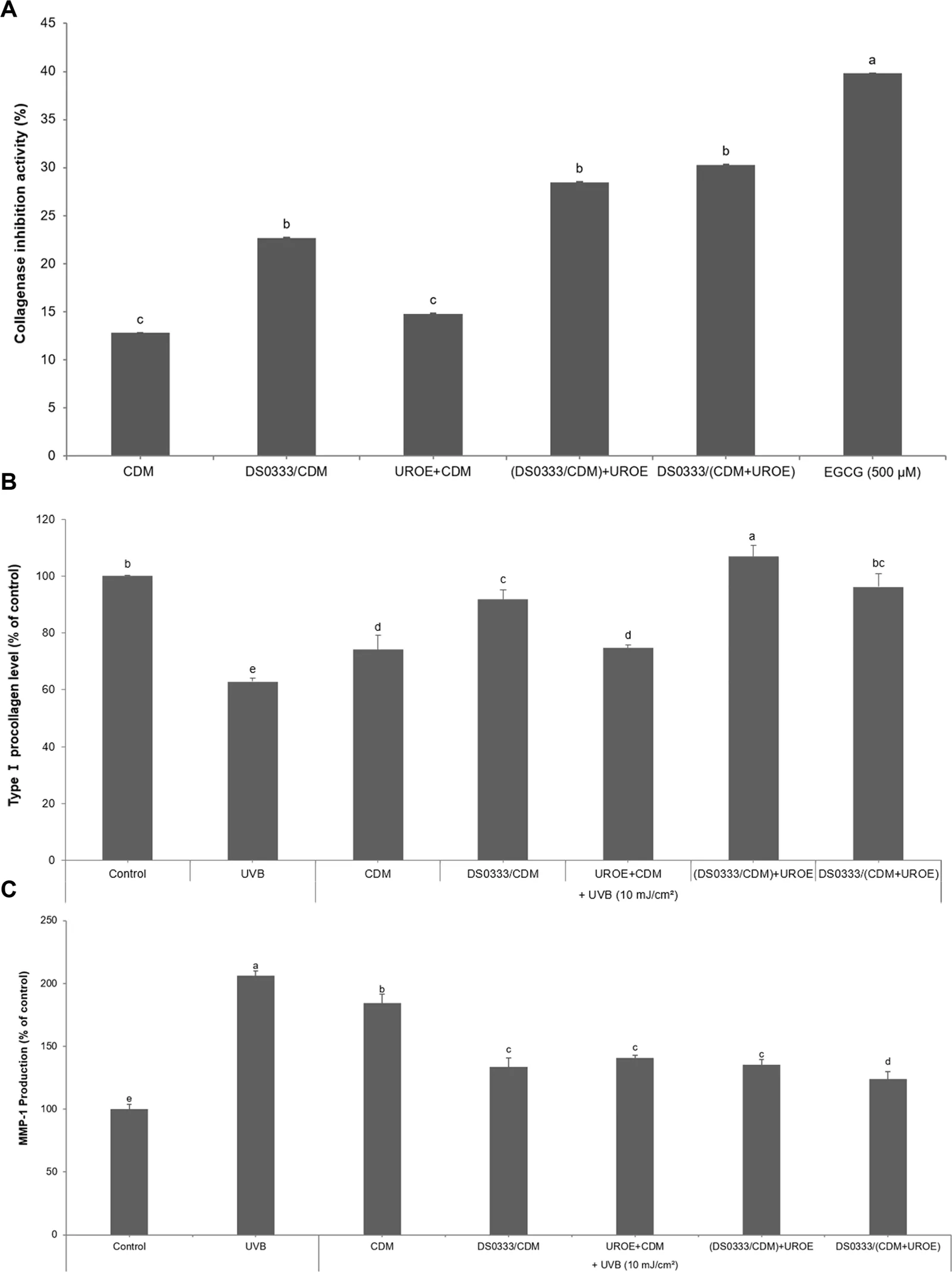
Anti-photoaging effects of the experimental samples. (**A**) Collagenase-inhibitory activity of the experimental samples. Epigallocatechin gallate (EGCG; 500 μM) was used as a positive control. (**B**) Type I procollagen production and (**C**) matrix metalloproteinase-1 (MMP-1) production in HDFs exposed to UVB irradiation (10 mJ/cm^2^) and treated with the indicated samples for 72 h. The levels of type I procollagen and MMP-1 are expressed relative to those of the untreated control, which were set to 100%. Data are presented as the mean ± SD of three independent experiments. Statistical differences were analyzed using one-way ANOVA followed by Duncan’s multiple range test. Values labeled with different lowercase letters are significantly different (*p* < 0.05).

**Fig. 7:**
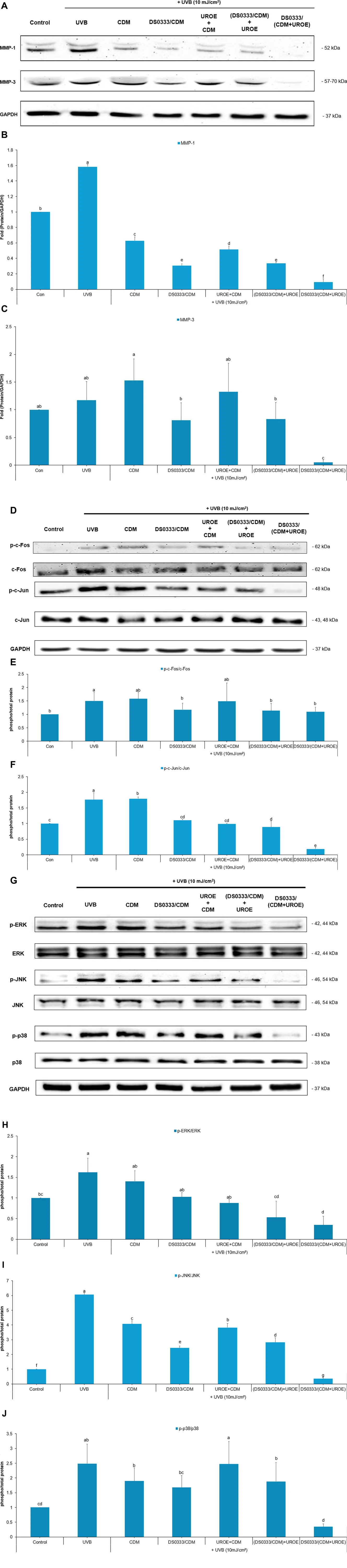
Effects of the experimental samples on MMP expression and AP-1/MAPK signaling in UVB-irradiated HDFs. (**A**) MMP-1 and MMP-3 were determined by western blotting, with GAPDH used as the loading control. Densitometric quantification of (**B**) MMP-1 and (**C**) MMP-3 expression was normalized to GAPDH and expressed relative to the untreated control. (**D**) Protein expression and phosphorylation levels of the activator protein-1 (AP-1) components c-Fos and c-Jun were determined by western blotting. Densitometric quantification of (**E**) phosphorylated c-Fos relative to total c-Fos and (**F**) phosphorylated c-Jun relative to total c-Jun. (**G**) Protein expression and phosphorylation levels of the mitogen-activated protein kinases (MAPKs) ERK, JNK, and p38 were determined by western blotting. Densitometric quantification of (**H**) phosphorylated ERK relative to total ERK, (**I**) phosphorylated JNK relative to total JNK, and (**J**) phosphorylated p38 relative to total p38. Representative western blot images are shown. Data are presented as the mean ± SD of three independent experiments. Statistical differences were analyzed using one-way ANOVA followed by Duncan’s multiple range test. Values labeled with different lowercase letters are significantly different (*p* < 0.05).

**Fig. 8:**
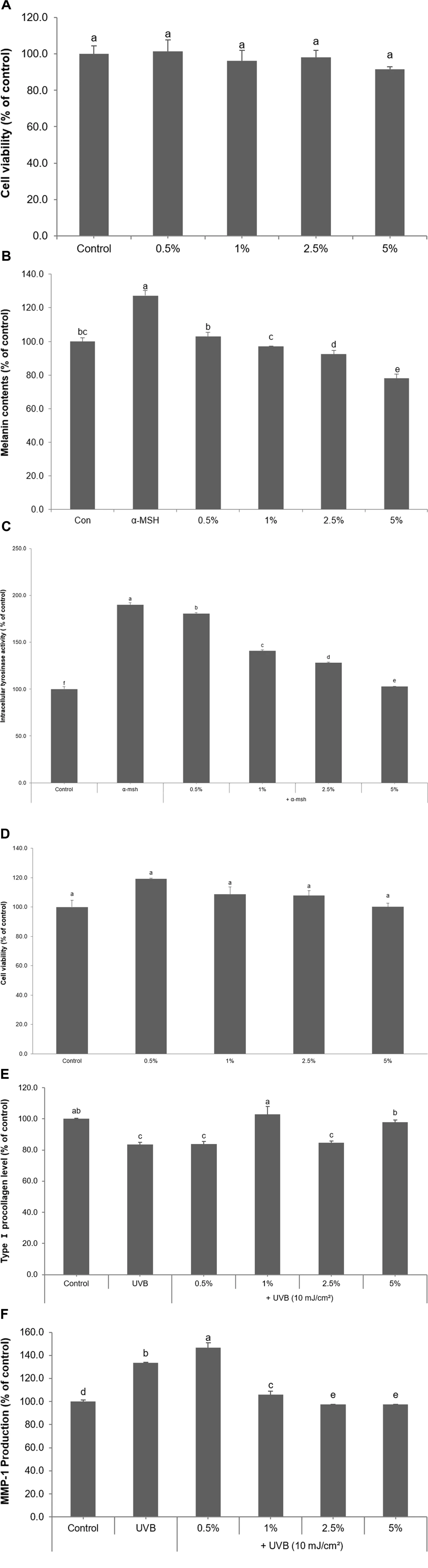
Concentration-response effects of DS0333/(CDM+UROE) on cell viability, melanogenesis, and photoaging-related parameters. DS0333/(CDM+UROE) was evaluated at concentrations of 0.5, 1, 2.5, and 5% (v/v). (**A**) Viability of B16F10 melanoma cells (**B**) Melanin content and (**C**) intracellular tyrosinase activity in B16F10 cells stimulated with α-MSH. (**D**) Viability of HDFs. (**E**) Type I procollagen production and (**F**) MMP-1 production in HDFs. All endpoints were evaluated after 72 h of treatment. Results are expressed relative to those of the respective untreated controls, which were set to 100%. Data are presented as the mean ± SD of three independent experiments. Statistical differences were analyzed using one-way ANOVA followed by Duncan’s multiple range test. Values labeled with different lowercase letters are significantly different (*p* < 0.05); values sharing the same letter are not significantly different.

**Table 1:** Definition of culture conditions and composition for each experimental group.

Group	Figure label	Description
1	CDM	Use of the chemically defined medium developed by Koduru *et al.* (2022) as the medium control
2	DS0333/CDM	DS0333 inoculated at 3% (v/v) into 3 mL CDM; culture supernatant obtained after cultivation
3	UROE+CDM	UROE added to CDM at a final concentration of 100 μg/mL without DS0333 inoculation
4	(DS0333/CDM)+UROE	Simple mixture of DS0333 culture supernatant and UROE; UROE adjusted to a final concentration of 100 μg/mL immediately before treatment
5	DS0333/(CDM+UROE)	DS0333 inoculated at 3% (v/v) into 3 mL CDM supplemented with 1% UROE, corresponding to an effective UROE concentration of 100 μg/mL during cultivation; culture supernatant obtained after cultivation

**Table 2:** HPLC analytical conditions.

Column	Agilent Eclipse XDB-C18 (4.6 × 250 mm, 5 μm)		
Injection volume	10 μL		
Wavelength	210 nm		
Flow rate	1 mL/min		
PLA standard range	6.25−100 μg/mL (retention-time reference only)		
Mobile phase	(A) Water (containing 0.2% phosphoric acid), (B) Acetonitrile		
Gradient conditions	Time (min)	A (%)	B (%)
	0	100	0
	1	100	0
	5	0	100
	20	0	100
	23	100	0
	30	100	0

**Table 3:** Antibodies used for western blot analysis.

Target protein	Supplier	Catalog no.	Dilution
MITF	Cell Signaling Technology (USA)	#12590	1:1,000
TRP-1	Abcam (UK)	ab178676	1:10,000
TRP-2	Abcam	ab2211144	1:1,000
MMP-1	Santa Cruz Biotechnology (USA)	sc-58377	1:400
MMP-3	R&D SYSTEMS	AF513	1:600
p-c-Fos	Cell signaling	#5348	1:1,000
c-Fos	Cell signaling	#2250	1:1,000
p-c-Jun	Cell signaling	#3270	1:1,000
c-Jun	Cell signaling	#9165	1:1,000
p-ERK	Cell signaling	#9101	1:1,000
ERK	Cell signaling	#9102	1:1,000
p-JNK	Cell signaling	#4668	1:1,000
JNK	Santa Cruz Biotechnology	sc-571	1:400
p-p38	Cell Signaling Technology	#4511	1:1,000
p38	Santa Cruz Biotechnology	sc-7149	1:200
GAPDH	Enogen Biotechnology (USA)	E12-057	1:5,000
